# Eliminating the Heart from the Curcumin Molecule: Monocarbonyl Curcumin Mimics (MACs)

**DOI:** 10.3390/molecules20010249

**Published:** 2014-12-24

**Authors:** Dinesh Shetty, Yong Joon Kim, Hyunsuk Shim, James P. Snyder

**Affiliations:** 1 Center for Self–assembly and Complexity, Institute for Basic Science, Pohang 790-784, Korea; E-Mail: dinuchem@gmail.com; 2 Department of Chemistry, Emory University, Atlanta, GA 30322, USA; E-Mail: ykim357@emory.edu; 3 Department of Radiology and Imaging Sciences, Emory University, Atlanta, GA 30322, USA; E-Mail: hshim@emory.edu; 4 Winship Cancer Institute, Emory University, Atlanta, GA 30322, USA

**Keywords:** monocarbonyl analogs of curcumin, MACs, inflammation, cancer, infectious disease, anti-angiogenesis, NF-κB, TNF-α

## Abstract

Curcumin is a natural product with several thousand years of heritage. Its traditional Asian application to human ailments has been subjected in recent decades to worldwide pharmacological, biochemical and clinical investigations. Curcumin’s Achilles heel lies in its poor aqueous solubility and rapid degradation at pH ~ 7.4. Researchers have sought to unlock curcumin’s assets by chemical manipulation. One class of molecules under scrutiny are the monocarbonyl analogs of curcumin (MACs). A thousand plus such agents have been created and tested primarily against cancer and inflammation. The outcome is clear. *In vitro*, MACs furnish a 10–20 fold potency gain *vs.* curcumin for numerous cancer cell lines and cellular proteins. Similarly, MACs have successfully demonstrated better pharmacokinetic (PK) profiles in mice and greater tumor regression in cancer xenografts *in vivo* than curcumin. The compounds reveal limited toxicity as measured by murine weight gain and histopathological assessment. To our knowledge, MAC members have not yet been monitored in larger animals or humans. However, Phase 1 clinical trials are certainly on the horizon. The present review focuses on the large and evolving body of work in cancer and inflammation, but also covers MAC structural diversity and early discovery for treatment of bacteria, tuberculosis, Alzheimer’s disease and malaria.

## 1. Introduction

**Figure 1:**
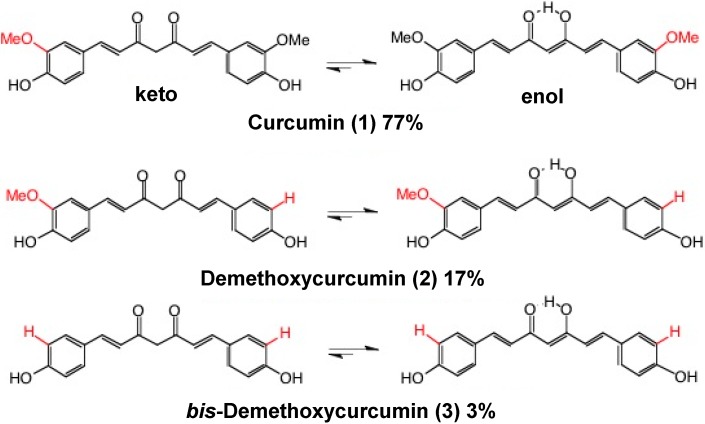
Curcumin and its demethoxy isomers isolated from turmeric.

Curcumin (**1**, diferuloylmethane, Figure 1) is an ancient and tantalizing molecule characterized by nicknames such as Indian Saffron, The Spice of Life and Indian Solid Gold. Extracted from fresh dried roots of *Curcuma Longa* and related species in the ginger family, it is distributed annually in over million ton quantities world-wide as the rough and heterogeneous extract “turmeric”, which contains over two hundred other natural small molecules. The mixture with 2%–8% curcumin can be refined to deliver both pure **1** and isomeric mixtures of the agent dominated almost entirely by the enol isomers (Figure 1). Many varieties of the natural product are popular primarily as food coloring and flavoring agents, spices, cosmetics, botanical supplements and medicines [1]. The internet is rich with the range of products available.

The medical history of turmeric and curcumin, particularly in Asia, is extensive and stretching from centuries-old traditional ayurvedic practice to modern times. In the current environment that combines medicinal chemistry, pharmacology, biochemistry and molecular biology, cucumin has surfaced as a pleiotropic agent able to interact directly or indirectly with a multitude of cellular proteins while appearing to exert a whole organism effect on an extensive range of human disorders. The literature includes claims that the molecule can serve as an antioxidant, antimicrobial, antifungal, antiinflammatory and wide-ranging anticancer agent. In the latter category, it has been reported to elicit benefits in connection with drug-resistance and metastasis. The extended list includes protection for heart ailments, arthritis, wound healing, depression and Alzheimer’s disease among many others. It is not surprising, then, that considerable health care research has been devoted to testing the efficacy of curcumin as a pure agent, in various formulations and in combination with other proven drugs. In the 2013–2014 time frame, the NIH reported over 90 clinical trials with curcumin integral to the therapy under investigation [2]. Yet no single curcumin-containing agent has been approved by the FDA. One possible reason could be the limited opportunity for protection of such a compound in an aggressive marketplace and a historical geographical context. In 1995, two researchers at the University of Mississippi (UM) sought and won a patent for curcumin’s ability to heal wounds. They also garnered the exclusive right to market turmeric. Within two years the Indian government’s Council of Scientific and Industrial Research protested the patent as biopiracy and challenged its novelty by showing that wound-healing is an ancient practice supported by equally ancient Sanskrit documents. Needless to say, the patent was revoked and India’s “national molecule” was rescued from exploitation by UM and its faculty [3].

In parallel with recent research on parent curcumin, many laboratories around the globe went in search of easily prepared novel agents with biological properties similar or superior to those of curcumin. A major chemical class, the monocarbonyl analogs of curcumin (MACs) evolved and is the focus of this review.

**Figure 2:**
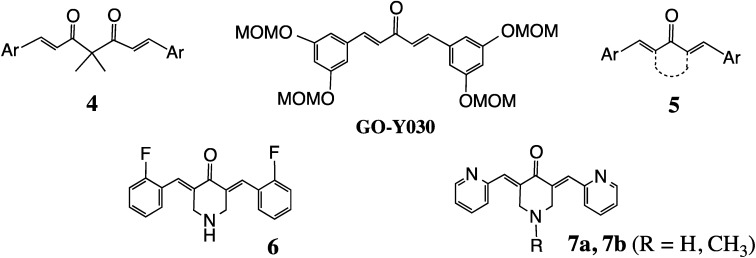
Curcumin mimics. FLLL series (**4**), GO-series (GO-Y030), MACs as acycle or ring (**5**), EF24 (**6**), EF31 (R = H, **7a**), UBS109 (R = Me, **7b**).

One might conclude that the driving force for this curcumin re-direction arose from the patent conflict between UM and India. However, a number of other crucial factors have been at work. That most often quoted is the meager bioavailability of the drug in humans resulting from aqueous insolubility, low absorption, rapid metabolism, poor chemical stability and fast systemic elimination [4] These considerations noted in the overwhelming majority of MAC papers cited herein imply the molecule to be less tantalizing as a drug candidate than its ancient legacy might otherwise suggest. Influential structural modifications of curcumin that improve stability and solubility involve elimination of the hydrolysis-prone keto-enol functionality in **1**–**3** [5,6,7,8] and incorporate a range of alternative substituents on the terminal phenyl rings. Two such replacements involve dialkyl substitution of the hydrogens on the carbon between the two carbonyl groups in the diketo tautomer (the FLLL family, **4**, Figure 2) [9] or installation of a single carbonyl group either as an acyclic agent or embedded in a small ring (the MAC family) (**5**, Figure 2). Both avoid the extraordinarily rapid decomposition of curcumin at pH 6.5 and above in aqueous medium [10] and deliver improved pharmacokinetic profiles in mouse models [11,12,13,14]. Enhancement of solubility is likewise readily achieved by appropriate substituent modification of MAC structures, the acyclic GO-series represented by GO-Y030 (Figure 2) [15,16,17] and combination of pyridines and piperidines such as EF24 (**6**) [18] and UBS109 (**7b**) [19] providing excellent examples. Accordingly, such molecular structures have attracted interest as models for development of novel curcumin mimics. On the other hand, not all of the natural product’s liabilities are bypassed by structural modification. MACs such as **6** and **7b** like curcumin [4,20,21], experience rapid reductive metabolism and generate metabolites that carry only a fraction of the activity of the fully unsaturated parent compounds [19].

Other reasons for turning from **1** to MACs are ease of synthesis [22,23] selectivity [9,24,25] and recognition that the pleiotropic nature [26] of the curcumin-like architecture permits rapid evaluation of drug potential for currently troublesome disorders such as highly resistant bacteria, Alzheimer’s syndrome, HIV, tuberculosis, malaria and diabetes [22,27]. Several excellent reviews detailing the diversity, applications and biological foundations of MACs for utility in human disease have appeared in the recent past [10,22,27,28,29,30,31,32].

## 2. Structural Diversity

### 2.1. 2D Diversity

**Figure 3:**
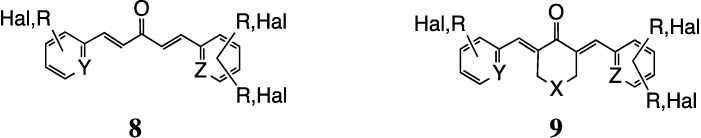
Core structures representing the diversity of individual MAC derivatives.

A casual survey of both reviews of the monocarbonyl curcumin literature and the vast collection of supporting peer-reviewed research papers reveals hundreds of variations on the theme created by elimination or restructuring of the keto-enol moiety in curcumin. Nonetheless, almost all of the individual compounds can be clustered into two core templates in the diarylpentanoid class of molecules, namely the acyclic form **8** and the cyclic variation **9** (Figure 3).

The majority of analogs are symmetrical consistent with ease of synthesis, however, many asymmetric versions have been prepared including chalcone variants [28,33]. The terminal aromatic rings may contain up to three different substituents and one or more nitrogen atoms in the ring to deliver heteroaromatic pyridine analogs (**8**, **9**, Y = Z = N) with N located at *o*, *m* and *p* sites. The most popular phenyl ring substituents are OR and OH followed by halogen atoms. However, N- and C-linked substituents have been probed as well. The terminal phenyls have also been replaced with heteroaromatic rings such as thiophene in a few cases. The central ring in **9** most often appears as a 5- or 6-membered ring, although a number of 7-membered ring congeners are known. The central 6-ring is often accompanied by X = C, NR, O, S and SO_2_. Surprisingly, no B-R or Se derivatives have appeared to date. Finally, the central carbonyl group is the overwhelming favorite functionality at its position, although C=NOH, C=C(CN)_2_ and related substances have been prepared. A sampling of individual structures representing MAC diversity is presented along with their biology below.

### 2.2. 3D Diversity

**Figure 4:**
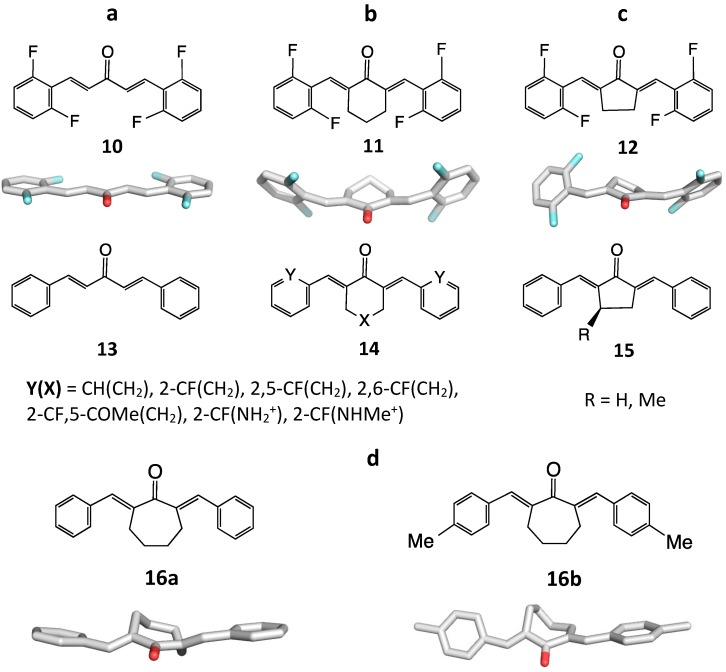
Five X-ray crystal structure geometries of selected diarylpentanoid MACs. In the structures shown, the aromatic rings are each substituted with fluorine at the *ortho*-postions C2 and C6 or without substituents; (**a**) Acyclic series exhibiting planar geometry; (**b**) Central 6-membered ring with an approximate plane of symmetry bisecting an envelope conformation; (**c**) Central 5-membered ring with a twist ring conformation; (**d**) 7-membered ring derivatives presenting two different conformations of the central ring.

Apart from the topological and structural variations described above, each of the mono-carbonyl analogs adopts a unique 3D structure in the solid state and a corresponding conformational profile in solution. The implication is that the delicate requirements for a molecule to bind and influence the behavior of a target protein will be dependent on the 3D geometry of the ligand. Thus, 2D representations such as **10**–**16** imply an undeserved similarity in terms of their complementarity to a chiral protein pocket. This is illustrated with the X-ray crystal structures of a small subset of MACs, several of which are substituted with fluorine as a rather diminutive replacement for hydrogen (Figure 4).

The structures depicted in Figure 4 reveal four separate 3D motifs. Acyclic fluorinated **10** [34] and the unfluorinated analog **13** [35,36,37] are both fully planar. Nonetheless, a polymorphic form of **13** [38] and a number of substituted analogs [38,39,40,41] adopt a twisted shape. Many MAC entries in the Cambridge Structural Database (CSD) show distortions from planarity, but the great majority involve metal complexation to one or both of the C=C double bonds. In the uncomplexed acyclic cases, *ortho*-fluoro substitution of the terminal phenyl rings does not perturb planarity. However, larger *ortho*-groups, e.g., CF_3_ or *i*-Pr, accompanied by steric hindrance will certainly induce non-planarity. Introduction of a 6-membered central ring, on the other hand, produces a butterfly shape as in **14** regardless of the nature of X (C, N, O or S) [42,43,44,45,46,47,48,49,50,51]. Interestingly, a variety of structural modifications including the formation of a nitrogen heterocycle, neutral or charged (Figure 4b, variations of X and Y), results in a very similar conformation verified by molecular superposition of the structures. One apparent exception to this observation is the cationic N-dimethyl analog **14** (X = NMe_2_^+^, terminal phenyl rings carry *p*-NMe_2_) [52]. The six-membered ring is a distorted half-chair, while the distal phenyl rings are twisted away from the butterfly shape. The 5-membered variant **12** (Figure 4c) adopts a highly unsymmetrical structure resulting from the adoption of a twist conformation by the central ring [42]. The X-ray structures of a family of nearly 20 analogs of the corresponding unfluorinated analog **15** (R = H) lacking *ortho*-substituents on the phenyl rings are fully planar similar to **10** and **13** [53]. Thus, it appears that internal steric effects occasioned by the four *o*-fluorine atoms in **12** is the basis for the observed asymmetric non-planarity. Other more bulky *ortho*-substituents can be expected to enhance the effect. A complement is the structure of **15** (R = Me) [54]. This molecule likewise exhibits a twisted 5-membered ring conveying both modest non-planarity and asymmetry to the overall molecular shape. Within the same MAC family, **15** (R = H) has recently been isolated as two different but nearly superimposable conformations representing a second polymorph of the compound. The origin of the two conformers and the new polymorph has been ascribed to C-H---O, π-π and C-H---π interactions [55]. Two 7-membered ring analogs (**16a**,**b**, Y = CH, X = CH_2_-CH_2_, Figure 4d) reveal yet other geometrical options in the form of two different conformers for the central ring and novel positioning of the terminal phenyl groups [56].

The message of this analysis is that 2D representations of MACs decorated with a range of substituents provide an incomplete picture of the fundamental nature of the interactions between potential drugs and their protein targets. Furthermore, the X-ray structures discussed above still reveal only the “tip of the iceberg”. The molecular shapes captured by small molecule crystal structures do not necessarily represent those for the same molecule bound to a protein. In solution, ligand molecules are properly described by an ensemble of conformations, one of which is likely to match the conformer within a protein pocket. It has been shown that low population conformers (<20%) are often the favored structure [57,58,59]. In the MAC context, head-to-head comparisons of the bio-potencies of 5- and 6-membered ring analogs occasionally reveal sharp differences most likely due to a combination of substituent effects, overall molecular shape and the conformation that is selected for binding to specific targets (Figure 3). Unfortunately, this makes SAR development complicated and reasoned molecular design difficult.

One recent study concerned with the interaction of pleiotropic MACs with kinases has nonetheless attempted to illustrate that the binding of similar core structures is tempered by both molecular shape and specific substitution pattern [26]. EF31 (**7a** and **14**, Y = N, X = NH) was used to screen a 50-member kinase library, and the top 12 enzymes were then subjected to IC_50_ inhibition measurements with five MACs. AKT1 and AKT2 delivered the lowest range of values (IC_50_ 0.02 to >100 μM) followed by a kinetic analysis to strongly suggest that the dominant mechanism for inhibition is competitive displacement of ATP. Accordingly, a structure-based analysis performed with AKT2. The top hit, N-protonated EF31 (IC_50_ 0.02 μM) was subsequently docked into the ATP binding and shown to make hydrogen bonds from its carbonyl group (CO---HN(CH_2_)) and one of the pyridine nitrogens, a salt bridge to Glu236 (N-H---OC(O)) and several attractive hydrophobic contacts. Comparison with protonated EF24 (**7a** and **14**, Y = C-F, X = NH_2_) with 40-fold lower potency (IC_50_ 0.8 μM) demonstrated loss of a key hydrogen bond and introduction of weaker ligand-protein associations. Protonated UBS109 (**7b** and **14**, Y = N, X = NMe), lowering potency still further (IC_50_ 1.9 μM), was predicted to relocate somewhat in the binding site to accommodate the slightly bulky equatorial N-methyl group. This movement not only created a pair of close steric contacts, but also eliminated the electrostatically enhanced C=O-HN(Lys181) hydrogen bond. The largest structure activity perturbation takes place with **7b**/**14** (X = S, Y = N; IC_50_ > 100 μM) relative to EF31 (**7a**) in which the central NH is replaced by sulfur, and the AKT2 potency drops by several thousand fold. Molecular modeling suggests a major relocation of the molecule in the binding pocket and the loss of two key electrostatically enhanced H-bonds (*i.e.*, from C=O and N-H). The relatively large sulfur atom and the expanded volume of the molecule due to its long S-C bonds are contributing factors. The reader is referred to the original literature [26] for additional details. One concludes that relatively small changes in drug-ligand structure, not necessarily apparent from comparison of flat textual structures (e.g., Figure 4), can have a drastic effect on the degree of binding with target proteins when they are known. In such circumstances, structure-based models can prove useful for understanding quantitative structure activity relationships (QSARs) and generating ideas for further synthesis and bioassay. In such cases, a ligand-based analysis can sometimes compensate for the lack of detailed structure. Unfortunately, in the sequel, most of the cases discussed do not yet allow a consideration of specific ligand-protein interactions. However, recognition that structural variation as illustrated in Figure 4 and the accompanying discussion, is operating beneath the cover of superficial structural comparison can alert one to expect both unintended frustration and surprises.

In this section, we have provided a consolidated structural introduction to be followed by a therapeutic organization, which brings together structure, mechanism and biology under specific disease/biology subsections. We have made an attempt to track disease indications associated with the range of structural modifications in the hope that this overview may serve as a useful basis for further analog development.

## 3. Inflammation Control *in Vitro* and *in Vivo* by MACs

**Figure 5:**
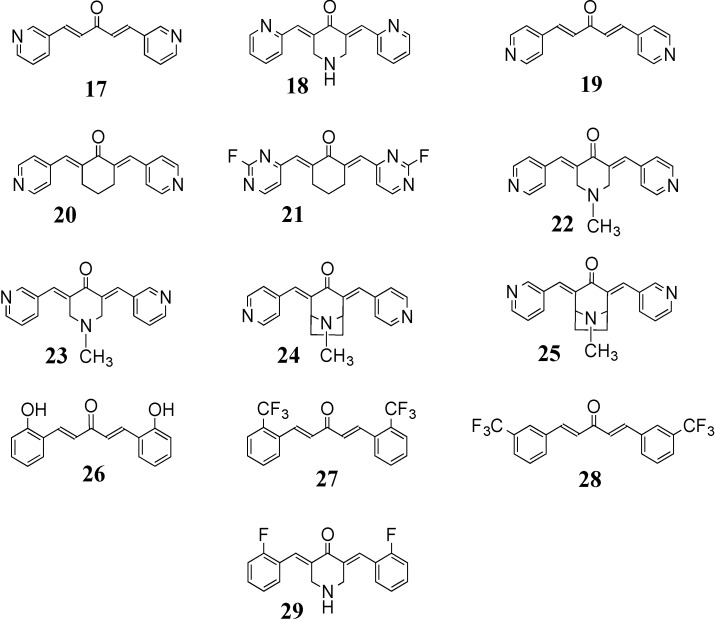
Structures of anti-inflammatory MACs **17**–**29**.

In general, the anti-inflammatory activity of curcumin analogs results primarily from inhibition of nuclear factor kappa-B (NF-κB), tumor necrosis factor (TNF)-α, and interleukin (IL)-6, NF-κB being a key transcriptional factor in the inflammatory signaling pathway. Many studies have reported that MACs may target both inflammation and tumors by inhibiting the activation of NF-κB [8,23,60,61,62,63,64,65,66,67,68,69,70,71,72,73,74]. The anti-inflammatory properties and the ability to inhibit the immune response by MACs, at least in part, result from inhibition of the activation of the latter multi-protein complex, since many of the genes that are implicated in the immune/inflammatory response are upregulated by NF-κB. MACs have also been shown to be a direct inhibitor of enzymes that are important in the inflammatory response, including lipoxygenase (5-LOX) and cyclooxygenase (COX-2) [75]. With few exceptions, most of the curcumin analogs with good anti-inflammatory action incorporate the diarylpentanoid linker instead of the β-diketone moiety and incorporate heteroatom and halogen moieties (**17**–**25**, Figure 5). Parallel studies have also confirmed that these analogs exhibit better anti-tumor, anti-inflammatory and anti-oxidant activity relative to curcumin (**17**–**19**, **26**–**29**, Figure 5).

### 3.1. NF-κB/TNF-α

Our group has synthesized two structurally similar analogs, 3,5-*bis*-(2-fluorobenzylidene)-4-piperidone (**6**, EF24) and 3,5-*bis*-(2-pyridinylmethylidene)-4-piperidone (**7a**, EF31) and compared their NF-κB inhibition activities in mouse RAW264.7 macrophages [61] Results showed that **7a** (IC_50_ ~ 5 μM) exhibits significantly more potent inhibition of lipopolysaccharide (LPS)-induced NF-κB DNA binding compared to both **6** (IC_50_ ~ 35 μM) and curcumin (IC_50_ > 50 μM). Compound **7a** also effectively blocks NF-κB nuclear translocation and the induction of downstream inflammatory mediators including pro-inflammatory cytokine mRNA and protein (TNF-α, IL-1β and IL-6). Furthermore, **7a** (IC_50_ 1.9 μM) shows significantly greater inhibition of IkB kinase β compared to **6** (IC_50_ ~ 131 μM). In addition to these efforts, Vileker *et al.* conducted a series of studies demonstrating the effectiveness of **6** to block mRNA synthesis of NF-κB dependent inflammatory factors [64].

**Figure 6:**
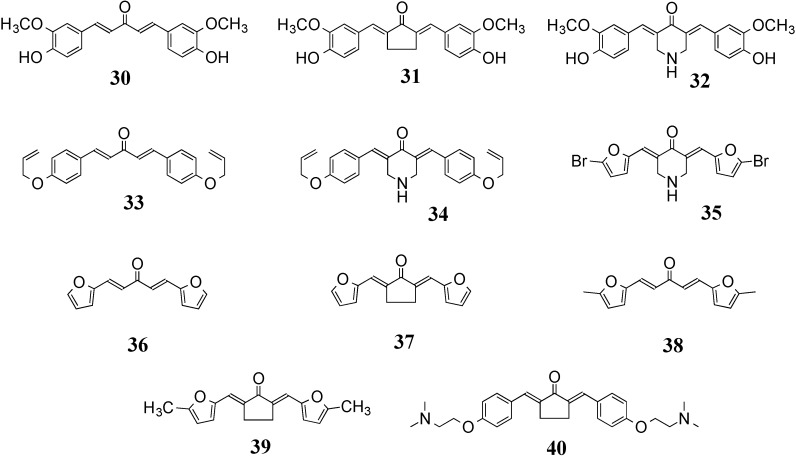
Structures of anti-inflammatory MACs **30**–**40**.

**Figure 7:**
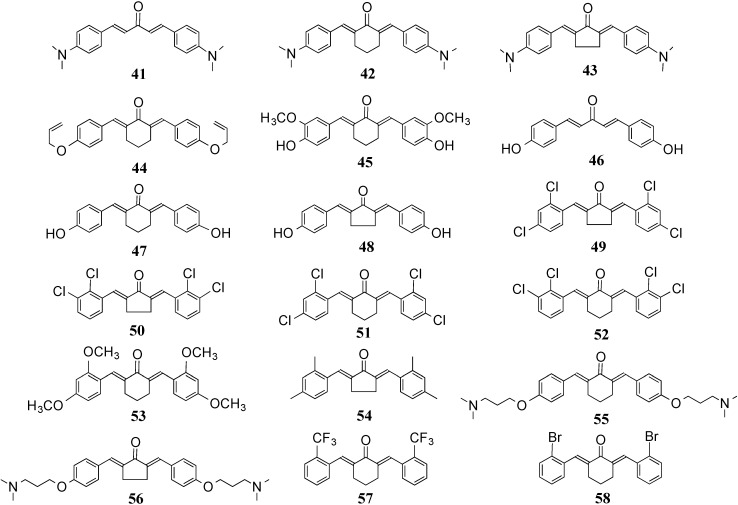
Structures of anti-inflammatory MACs **41**–**58**.

Liang *et al.* reported a series of MACs (**30**–**40**, Figure 6) with the ability to inhibit LPS-inducing macrophages that release inflammatory cytokines TNF-α and IL-6 via *in vitro* cell experiments [65,66]. Systematic structure-activity relationship studies on these compounds showed that multiple analogs can block expression of the inflammatory factors. Cyclohexanone-containing derivatives are somewhat more effective than acetone or cyclopentanone-derived compounds [65]. Installation of a long chain substituent such as 3-(dimethylamino) propoxyl (compound **40**) shows an inhibitory effect on LPS-induced TNF-*α* expression similar to curcumin, but a more potent inhibitory effect on LPS-induced IL-6 expression than curcumin. However, the dimethylamino-analogs **41**–**43** (Figure 7) exhibit either similar or reduced inhibitory effects on LPS-induced TNF-*α* or IL-6 expression relative to curcumin, indicating that nitrogenous substitution by itself does not enhance anti-inflammatory activity. On the other hand, **33** and **44** (Figure 6 and Figure 7) with a long chain allyloxyl moiety shows a stronger inhibitory effect on LPS-induced TNF-*α* indicating that the length and flexibility of the distal substituents may be favorable to the anti-inflammatory activity.

Among curcumin-like compounds, **30**, **31**, and **45** (Figure 6 and Figure 7) deliver the best inhibition activities while **46**, **47**, and **48** (Figure 7) are essentially inactive, suggesting that the presence of a 3-methoxy group in combination with the 4-OH group is critical to activity. The electronegative property of a substituent in the 4’-position plays an important role in anti-inflammatory activities [66]. Compounds without a *para* substituent in the phenyl rings show little inhibitory activity, whereas the presence of electron-withdrawing chloro substituents removes the anti-inflammatory activity completely (**49**–**52**, Figure 7). By comparison, *tetra*-methoxy **53** or *tetra*-methyl **54** (Figure 7) with 4’-substitution showed significant inhibitory activities against LPS-induced TNF-α and IL-6. These results indicate that the anti-inflammatory activity induced by LPS may be associated with the electronegativity of 4’-substituents. Electron-donating capacity from this position may increase the anti-inflammatory abilities, whereas a neutral and electron-withdrawing moiety may reduce or remove such bioactivity. Among all the compounds studied, **40** and **44** showed the highest potential as anti-inflammatory agents. However, the underlying molecular mechanisms of N-substituted long-chain substituents at the transcriptional or post-transcriptional levels are yet to be determined. One possible origin of the substituent-influenced inflammatory variation may be the enhanced resonance interaction of electron-donating functionalities with the enone group so as to attenuate its electrophilic properties. Since thiol conjugation appears to be a critical feature for biologically active enones [76,77] perturbation of the Michael addition for MACs by *para*-substituents may have a decisive influence on the degree of anti-inflammatory action. In mouse primary peritoneal macrophages, **55** (Figure 7) potentially inhibited the production of pro-inflammatory gene expression including TNF-a, IL-1b, IL-6, iNOS, COX-2 and PGE synthase. This activity was ascribed in part to the inhibition of ERK/JNK phosphorylation and NF-κB activation. Compound **55** likewise shows significant *in vivo* effects on pro-inflammatory cytokine production in plasma and liver; namely, attenuated lung histopathology and reduced mortality in endotoxemic mice [67]. In LPS-challenged mice, pretreatment by **56** (Figure 7) attenuated the increase of plasma levels of NO, TNF-α and IL-6, while significantly reducing the hepatic inflammatory gene transcription by the inhibition of various inflammatory mediators [68]. Compounds **57** and **58** (Figure 7) significantly alleviate renal and cardiac injuries in diabetes mellitus by means of an anti-inflammatory mechanism [69,70]. Further studies revealed that these anti-inflammatory actions are mediated by inhibiting the JNK/NF-κB signal pathway [67,69,71]. The Liang group has reported that, in general, the six-membered cyclohexanone ring system (IC_50_ values from 4 to 180 µmol) is superior to the five-membered cyclopentanone system (IC_50_, 1 to 222 µmol) for inhibitory activity [8]. The difference is often modest, but can be significant depending on the cell line used for the comparison. Some of analogs screened by Liang *et al.* have undergone preclinical study for the treatment of arthritis, pyemia (multiple abscesses caused by pus-forming microorganisms) and nephritis (kidney inflammation).

**Figure 8:**
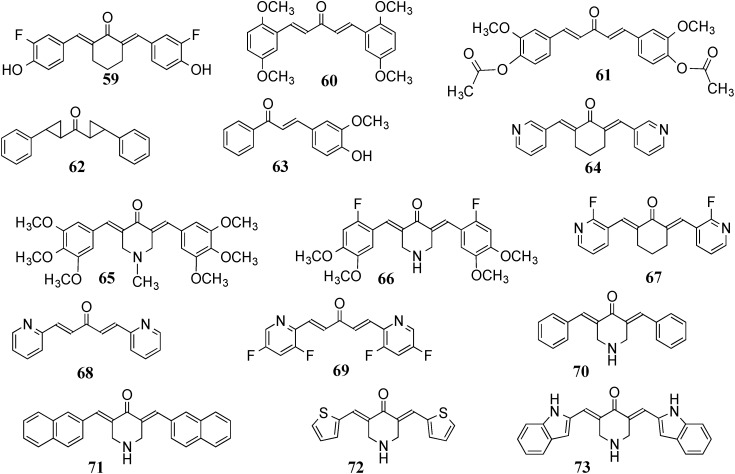
Structures of anti-inflammatory MACs **59**–**73**.

Weber *et al.* studied the inhibition of TNFα-induced activation of NF-κB by dienone MACs (**17**, **19**, **27**, **28**, **59**, Figure 5 and Figure 8) by using the Panomics’ NF-κB Reporter Stable Cell Line [72]. The enones tested included analogs with both a 5-carbon spacer (**17**, **26**, **30**, **60**–**62**, Figure 5, Figure 6 and Figure 8) and a 3-carbon spacer (**63**, Figure 8) separating the aromatic rings. The former are highly active and include cases with heterocyclic rings such as **17** (IC_50_ = 3.4 µM), the most active agent among the tested enones. While these compounds retain the enone functionality, their NF-κB inhibition does not correlate with their anti-oxidant activity. The study revealed that prevention of stress-induced activation of NF-κB by MAC analogs was achieved by inhibition of specific targets rather than by an overall anti-oxidation process. The latter action by MACs depends on the ability to quench free radical reactions, but can be complemented by inhibition or inactivation of specific targets. Researchers have tested the abilities of MACs to quench the pre-formed radical monocation of 2,2'-azinobis-(3-ethylbenzothiazoline-6-sulfonic acid), known as the total radical-trapping anti-oxidant parameter (TRAP) assay, and their ability to reduce the ferric tripyridyltriazine complex, namely the ferric reducing/antioxidant power (FRAP) assay [78]. Most active analogs probed in this study (**17**, **19**, **28**) show no activity in the TRAP or FRAP assay, which led to the conclusion that there is no correlation between anti-oxidant activity and inhibition of the TNFα-induced activation of NF-κB. This lack of correlation suggests that MACs inhibit specific targets rather than operate through redox chemistry.

Yadav *et al.* synthesized the series of heterocyclic cyclohexanone analogues **22**, **20**–**25**, **65**–**67** (Figure 5 and Figure 8) which were screened for inhibition of NF-κB transactivation in non-adherent leukemia cells [73]. The pyridine heteroaromatic substituted *bis*-methylene cyclohexanones **22** and **23** were the most active heteroaromatic analogues (IC_50_ = 1.0 µM and 0.8 µM), while the corresponding five-membered heteroaromatic agents exhibit considerably less activity (e.g., **36**, IC_50_ = 34 µM). The polymethoxyphenylmethylene N-methylpiperidone derivatives **66** and **67** (Figure 8) reveal good activity (IC_50_ = 0.9 and 4.0 µM, respectively) in the luciferase assay compared to the cyclohexanone core derivatives (~30 fold less activity), which had no activity. These results suggest that in addition to the electronic effects of substituents on the terminal aromatic rings described above, a nitrogen heteroatom in the aromatic rings or heteroatoms in the core cyclic ketone enhance potency. Cao *et al.* have reported pyridinyl analogs of dibenzylideneacetone (**68**, **69**, Figure 8), and these MACS were evaluated for their anti-inflammatory activity by the NF-κB inhibition assay in colorectal carcinoma cells [74]. Almost all synthesized analogs exhibited better cytotoxicity than curcumin, and **68** in particular delivered the highest anti-NF-κB (IC_50_ = 0.52 µM) potency in suppressing growth of colon cancer cells (IC_50_ = 1.6 µM).

**Figure 9:**
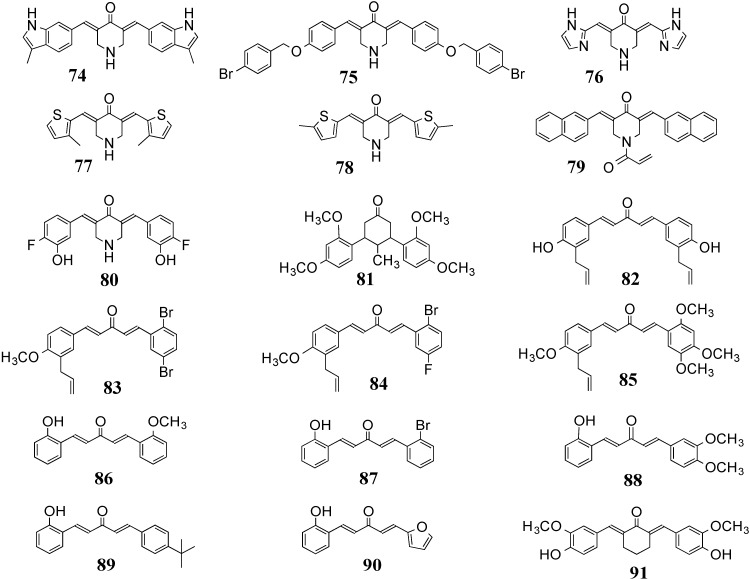
Structures of anti-inflammatory MACs **74**–**94**.

Recently, Liu *et al.* synthesized a series of allylated or prenylated MACs and evaluated their anti-inflammatory effects in RAW 264.7 macrophages [79]. A majority of the compounds effectively inhibited the LPS-induced expression of TNF-α and IL-6. The preliminary and quantitative SAR analyses showed that the asymmetric MACs possess higher anti-inflammatory activity than symmetric analogs, and suggested that the electronegativity and molecular polarizability of the MAC structures are important for the inhibition of LPS-induced IL-6 expression. Among the tested compounds, **82**–**85** (Figure 9) exhibited stronger inhibition of both TNF-α and IL-6 than curcumin. In particular, **83** delivered the most potent effects with inhibitory rates reaching 68% and 91%, respectively, and exhibited significant protection against LPS-induced death in both septic mice models and primary peritoneal macrophages. A related and recent report described the synthesis and evaluation of various asymmetric MACs as anti-inflammatory agents by inhibiting the LPS-induced secretion of TNF-α and IL-6. Among the test subjects, **86**–**90** exhibited dose-dependent inhibition. The anti-inflammatory activities of analogs **86** and **87** were associated with inhibition of the phosphorylation of the extracellular signal-regulated kinase ERK and the activation of NF-κB [28].

### 3.2. 5-LOX/COX-2

Cyclovalone (**91**) and three analogs (**92**–**94**) incorporating the diarylpentanoid linker between the terminal phenyl rings show anti-COX activity. The dimethylated analogs **92** and **94** are more potent than **95** and **97** that are, in turn, more potent than curcumin, suggesting the addition of methyl groups on the phenyl rings enhances anti-COX activity [80].

A series of MACs **70**–**79** (Figure 8 and Figure 9) containing a variety of heterocyclic rings (the indolyl, imidazolyl and thienyl rings) was synthesized by Katsori *et al* [75] and investigated for their ability to inhibit the inflammation related enzymes 5-LOX and aldose reductase (ALR2). Results of these studies revealed that **71** and **79** are more potent inhibitors of inflammatory enzymes 5-LOX and ALR2 than curcumin. Compounds **75** and **76** containing bromobenzene or heterocycles exhibit a high *in vivo* anti-inflammatory activity assessed by using the functional model of carrageenin-induced rat paw edema (expressed as percent inhibition of carrageenin-induced inflammation), while **75** showed much higher efficacy than indomethacin.

Gafner *et al.* designed and synthesized MACs (**59**, **80**) for inhibition of COX-1 and COX-2 and tested them in murine macrophages. Fluoro substitution in the MACs cyclohexanone analog **80** (Figure 9) enhanced anti-inflammatory activity, while nitro and *tert*-butyl substitution decreased it [81]. Based on COX-2 inhibition, **59** (IC_50_ = 5.5 µM) is more effective than other derivatives as well as curcumin (IC_50_ = 15.9 µM). The data suggests that structural elements responsible for COX-1 and COX-2 inhibition do not correlate well with those responsible for inhibiting COX-2 and iNOS gene expression. However the same elements do contribute to inhibition of 12-O-tetradecanoyl-13-acetate (TPA)-induced ornithine decarboxylase (ODC) activity. TPA-induced ODC is a rate-limiting enzyme process in the polyamine biosynthetic pathway. Certain polyamines are known to be important for cell growth and differentiation and have been implicated in the early phase of tumor promotion. Thus, diminishing ODC activity has been used frequently as a marker for inhibition of tumor promotion. Weber *et al.* reports highlight MAC analogs **17** (IC_50_ 4.1 μM) and **81** (IC_50_ 3.8 µM) as potential COX-2 inhibitors, which imply an important role in pro-inflammatory stimulation via TPA-induced activation of AP-1 [82,83].

In general, the position and electronegativity of substituents on the terminal aromatic rings and the length of the spacer between these rings determine the anti-inflammatory activities of MAC’s. For example, bromo substitution at the 2-position (compound **58**) shows little activity, whereas substitution at the 3-position results in superior anti-inflammatory activity compared to curcumin (IC_50_ 10–20 µM). MACs incorporating the cyclohexanone moiety are reported to be slightly more effective than those with acetone and cyclopentanone as the central core of the molecules [84]. Analogs bearing a long chain allyloxy substituent (**40**, **55**, **56**) exhibit enhanced activity, while analogues with dimethylamino or trifluoromethane substituents reveal diminished activity. Analogs possessing 3-methoxy (**30**, **31**, **45**) or trimethoxy (**66**) substitution display higher activity than curcumin. Heteroaromatic ring-substituted MACs (**17**, **18**, **21**–**25**) also show moderate anti-inflammatory activity [65,72,83,84]. All of these effects have their counterparts in the action of MACs *in vitro* in tumor cells and *in vivo* in murine models. The complementary actions of ablating both inflammation and cancer have their origin in blocking the same set of cell signaling pathways. This phenomenon is explored in detail in the next section addressing the action of MACs in the cancer environment.

**Figure 10:**
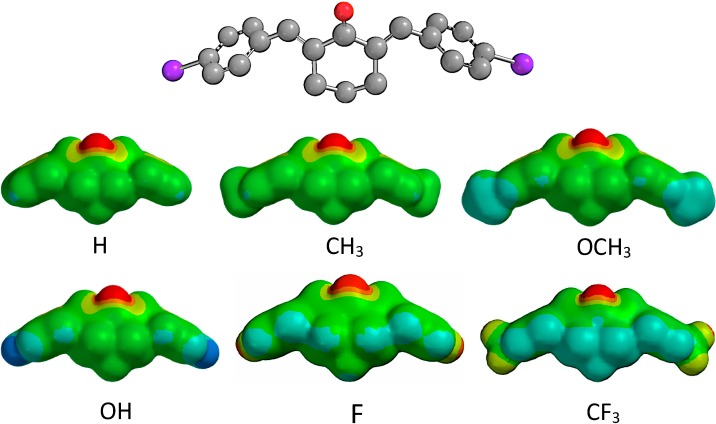
Electrostatic potentials for a series of piperidinone analogs with *para*-substituents H, CH_3_, OCH_3_, OH, F and CF_3_ situated at the purple centers; charges: green/neutral, red/negative and blue/positive; surfaces generated with the semiempirical PM3 method in Student Spartan [85].

The astute reader may note that several reports cited in this section highlight the influence of electronegative substituents at the *para*-positions of the MAC terminal aromatic rings as a mediator of anti-inflammatory activity. This point arises from the intuitive observation that electron-donating substituents appear to decrease inflammation, while electron-withdrawing groups are less effective or eliminate it [28,66,84]. The productive use of electronegativity descriptors in QSAR correlation treatments is consistent [79]. These works do not, however, provide a qualitative rationale for how differential electronegativity might be exerting the proposed effect. Prompted by a Reviewer, we propose the phenomenon to be complex and composed of several reinforcing forces. The following Figure 10 suggests the interplay of some of them.

For substituents H and CH_3_, apart from the C=O moiety, the molecular surface is neutral, but the methyl groups of OCH_3_ begin to exhibit slight positive charge in the molecular view shown. This is enhanced by OH substitution and further magnified across the entire molecular surface for F and CF_3_. These changes in electrostatic potential contribute to the binding of these molecules to the protein targets in the body’s cells. This is clearly not the whole, picture, however, since the protein binding pockets also need to accommodate the steric bulk of the *p*-substituents. In addition the OCH_3_, OH, F and CF_3_ groups must be compatible with any H-bonding or complementary electrostatic interactions of the MACs within reach at the binding center. Consequently, the charge distributions induced by the different electronegative atoms or groups partner with atomic size and non-bonded contacts to provide the maximum binding arrangement and, thereby, result in the associated ligand-protein affinities. These, in turn, undoubtedly modulate the strength of the ultimate anti-inflammatory output at the terminus of a long train of linked intra- and intercellular physiological events. For now, the latter remain a mystery yet to be unraveled.

## 4. Cancer Mediation *in Vitro* and *in Vivo* by MACs

### 4.1. In Vitro Probes of Cancer Cell Lines and Signaling Factors

The major actions of MACs reported in the anticancer literature are mainly anti-angiogenic and cytotoxic/anti-proliferative in the context of *in vitro* assays. These two phenomena correlate well, suggesting that the same signaling pathways or proteins are involved in the inhibitory processes. Not surprisingly, the same mechanistic factors may also be involved in the inflammatory events described in the previous section. However, unlike studies in inflammation research, investigators in cancer have most frequently evaluated MAC curcumin analogs in phenotypic assays, such as proliferation and angiogenesis, rather than mapping signaling pathways or identifying specific protein targets. Curcumin is, of course, the exception. A recent review on breast cancer by Cridge, Larsen and Rosengren summarizes associated molecular targets and points out the intervention of cytokines, growth factors, apoptosis and cell cycle proteins in addition to transcription factors and enzymes for a subset of MACs [32]. A recent modest kinase screen demonstrated that one MAC, EF31 (**7a** = **14**, X = NH, Y = N, Figure 2), is able to block 22 of 50 cancer-related kinases and suggests a mechanism dominated by competition with ATP [26]. Clearly more work needs to be done in this area, but it is highly likely that a majority of intracellular pathways established for curcumin will be followed by the MAC class of compounds, but with at least 10–20 fold greater potency.

**Figure 11:**
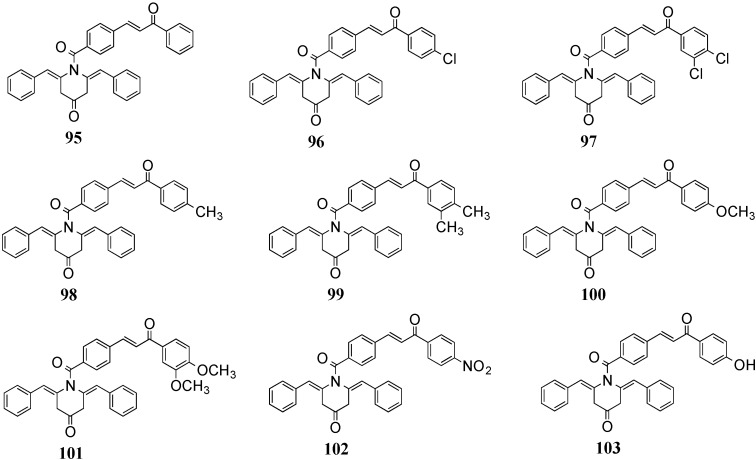
Structures of anti-cancer MACs **95**–**103**.

**Figure 12:**
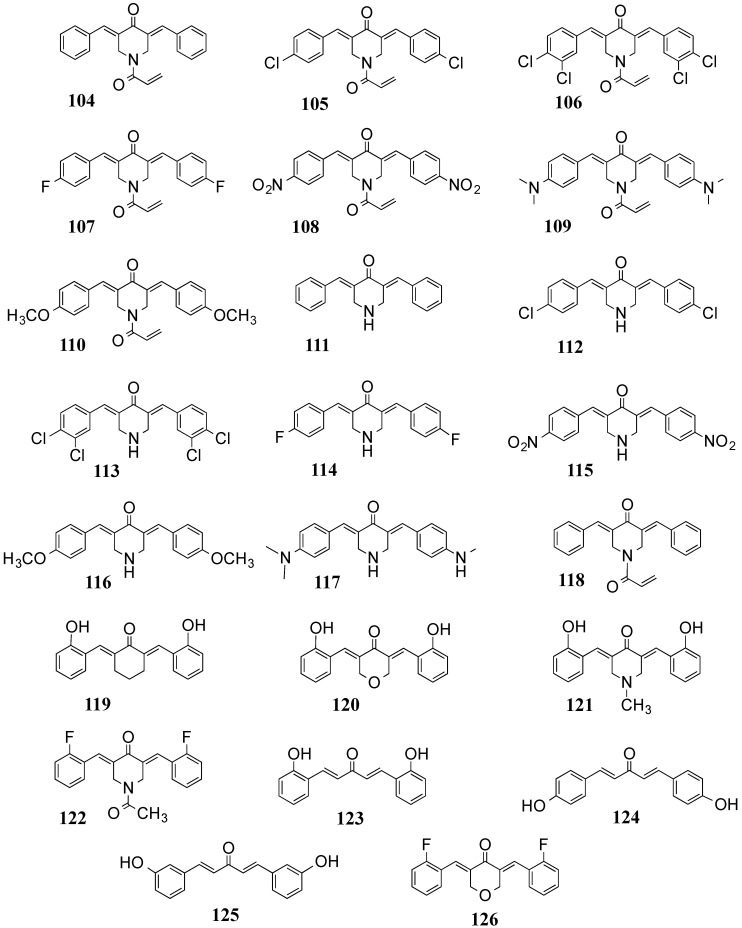
Structures of anti-cancer MACs **104**–**126**.

For example, Dimmock and colleagues synthesized a series of symmetric piperidones (**95**–**118**, Figure 11 and Figure 12) and used murine P388 and L1210 cells as well as human Molt4/C8 and CEM T lymphocytes to evaluate cytotoxic effects [86,87]. The average IC_50_ values for the N-acryloyl analogs **104**–**110** for the four cell lines was 1.8 μM, while the N-unsubstituted compounds **111**–**117** delivered a considerably higher average of 44 μM. Thus, within this series of substances, the N-substituted analogs furnish considerably greater cytotoxicity, and the SAR correlates positively with the size of the aryl substituents. However, in the three clusters of agents investigated, the SARs are specific to each series. Thus, for **111**–**117**, electronic parameters were deemed the most important factor influencing cytotoxicity.

Adams *et al.* synthesized a separate series of MACs and screened them for anti-proliferation and anti-angiogenic activity. Analogs **6** (EF24), **119**, **120** and **121**, among others, exhibited excellent cytotoxicity superior to that for cisplatin [23]. Those analogs effective in the anti-proliferation assay were also efficacious in anti-angiogenisis assays. For example, **6** is almost as potent as TNP-470, which has undergone clinical evaluation as an anti-angiogenic drug [23]. These data suggest: (1) The symmetrical α,β-unsaturated ketone moiety installed in the analogs shows increased anti-cancer and anti-angiogenesis activity compared with the β-diketone structure of curcumin; (2) *Ortho*-substitution on aromatic rings (**119**–**121**, Figure 12) in some cases enhances the activity for symmetrical analogs, the *meta*- or *para*-substitutions (**124**, **125**, Figure 12) being somewhat less active possibly due to alterations in molecular geometry (Figure 4); and (3) Introduction of a heteroatom in the cyclic ketone (**120**, **121** and **126**, Figure 12) generally yields improved anti-cancer and anti-angiogenic activity.

Compound **6** (EF24) was further studied by Thomas *et al.* and found to decrease cell viability of lung cancer cells via upregulated mitogen-activated protein kinases (MAPK) as evidenced by increased ERK1/2, c-Jun N-terminal kinase (JNK) and p38 (stress-activated protein kinase) phosphorylation [62]. A synergistic effect between the P38 inhibitor and **6** with respect to clonogenic activity of A549 lung cancer cells and apoptosis induction was also reported. Another interesting observation for **6** was revealed in relation to its anti-hypoxia inducible factor (HIF)-1 activity compared to curcumin. While curcumin inhibited HIF-1α gene transcription, **6** inhibited HIF-1α post-transcriptionally. The inhibition phenomenon occurred in a von Hippel Lindau (VHL)-dependent, but proteasome-independent manner. An additional difference was that curcumin induces microtubule stabilization in cells while **6** has no effect [88].

**Figure 13:**
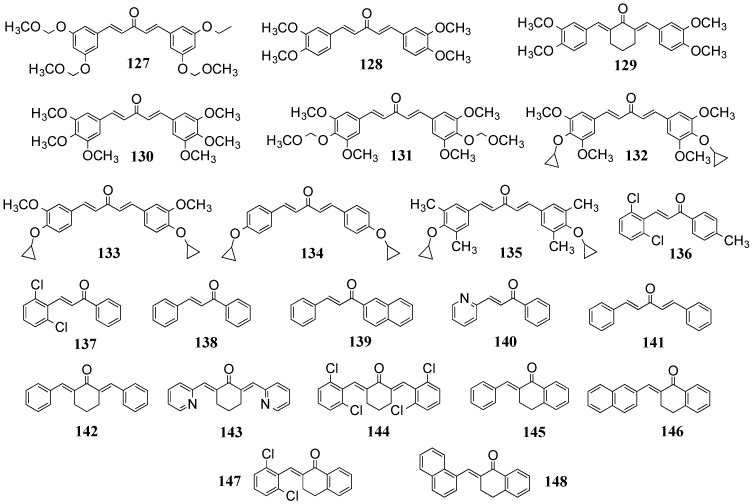
Structures of anti-cancer MACs **127**–**148**.

To evaluate MAC effects on colon cancer cells, Ohori *et al.* synthesized and screened **127**–**131** (Figure 13) against cell growth. The analogs are symmetrical 1,5-diarylpentadienones, the aromatic rings of which possess alkoxy substituents at *meta*- or *meta*/*para*-positions. Compound **128** was found to exhibit four-times higher potency than curcumin (IC_50_ 2 µM *vs.* 8 µM) [89].

Chandru and colleagues prepared the dienone cyclopropoxy curcumin analogs **132**–**135** (Figure 13) and evaluated the quartet by anti-proliferation and anti-angiogenic assays employing an *in vivo* Ehrlich ascites tumor mouse model. The agents significantly reduced ascite volumes accompanied by increased apoptosis. Anti-angiogenic activity was demonstrated by the significant reduction of microvessel density in the peritoneum wall sections. The study was interpreted to imply that the two aromatic regions might be critical for potential drug-protein interactions [90].

Aromatic enone and dienone analogues (**136**–**144**, Figure 13) were prepared by Robinson *et al.* and screened in an *in vitro* anti-angiogenic assay [91]. The compounds inhibited cell proliferation, **140** and **143** being particularly potent, suggesting the importance of heterocyclic substitution. The same group subsequently generated derivatives differing in either the substitution pattern of the benzene rings or the fusion characteristics of the aromatic rings. The most notable compounds **145**–**148** (Figure 13) are tetralones which introduce a measure of rigidity by tethering the central enone moiety. The 2-naphthyl analog **146** exhibits the best anti-angiogenic activity, 85% at 1 µg/mL in the following sequence: **146** > **147** > **145** > **148** [92].

**Figure 14:**
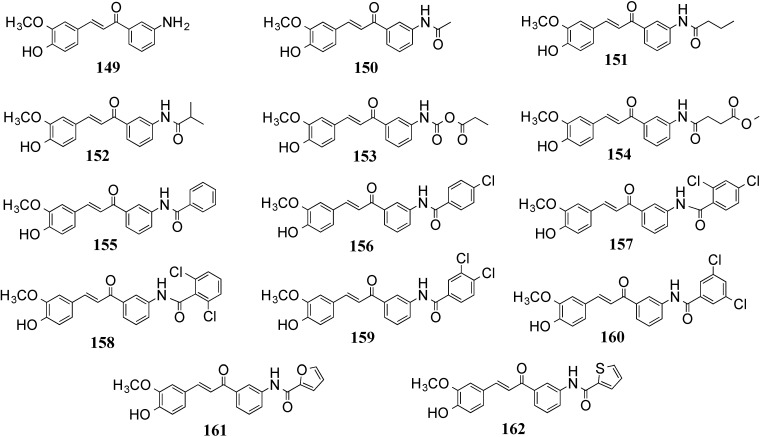
Structures of anti-cancer MACs **149**–**162**.

Woo *et al.* conceived a series of asymmetric MAC chalcones by pairing substituted phenyl amides with the terminal curcumin styrene unit carrying *m*-OMe and *p*-OH; *i.e.*, **149**–**162** (Figure 14). The *in vitro* growth inhibition of human umbilical vein endothelial cells (HUVEC) caused by **149**, **158**, **161** and **162** reflects potent anti-angiogenic activity and suggests it may be particularly important for asymmetric phenyl alkyl amides coupled with heteroaromatic moieties [93].

**Figure 15:**
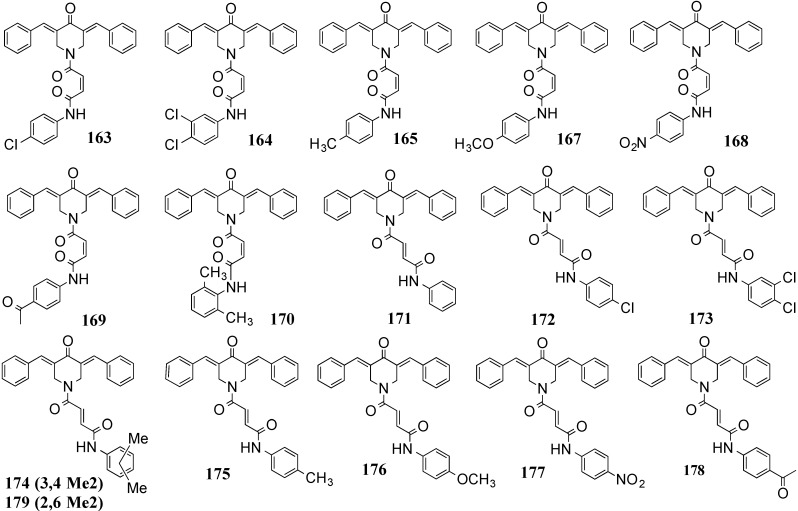
Structures of anti-cancer MACs **163**–**179**.

Jha *et al.* synthesized a series of highly polar MACs **163**–**179** (Figure 15) by replacing the unstable keto-enol moiety of curcumin with a substituted piperidone and testing the series against tumor inhibition activity [94]. In human Molt 4/C8 cells and CEM T-lymphocytes, compounds **171**–**179** were significantly more potent than the control agent melphalan [95] in inhibiting leukemia and colon cancer cell lines. However, compounds **163**–**170** lost activity. The change in potency is undoubtedly due to the geometric disposition of the double-bond configuration within the (O=C)-CH=CH-(C=O) moiety of the piperidone substituent; *i.e.*, *E vs*. *Z* stereochemistry. The most likely planar *E* form is expected to be twisted out of planarity in the *Z* isomer by steric effects encountered from the *syn* olefin orientation. The corresponding X-ray structures would offer deeper insights into this hypothesis, but in the spirit of the message of Figure 4, we presume that co-planarity of the compounds plays a key role in exerting cytotoxicity in the target proteins.

**Figure 16:**
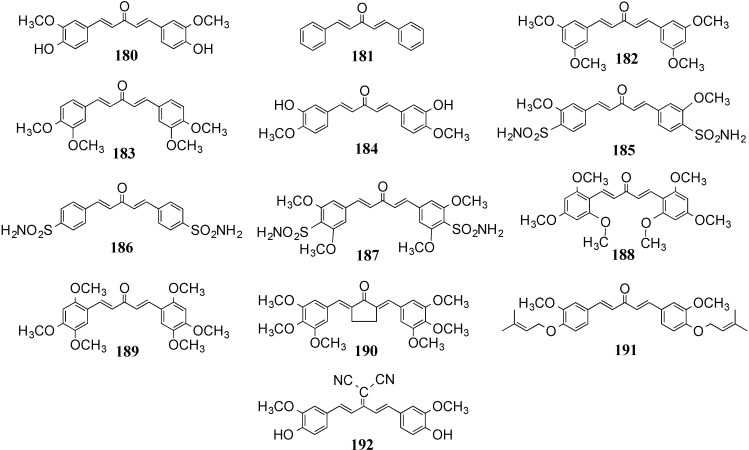
Structures of anti-cancer MACs **180**–**192**.

Fuchs *et al.* synthesized a collection of largely acyclic MACs **180**–**192** (Figure 16) and tested their anti-tumor properties by blocking the proliferation of prostate and breast cancer cells [96]. Compound **188**, decorated with three methoxy groups at *ortho* and *para* terminal ring sites, is particularly attractive with an IC_50_ within the 10^−6^ M range corresponding to an inhibitory potency of more than 50-fold higher than curcumin. Suarez and colleagues prepared a similar subset of compounds **61**, **180**, **181**, **191** and **192** (Figure 8 and Figure 16) and tested them against several tumor cell lines [97]. All the compounds exhibited different degrees of inhibitory activity against colon cancer cells HT-29, but analogs **61**, **180** and **181** furnished superior potency (IC_50_ < 2.3 μM).

**Figure 17:**
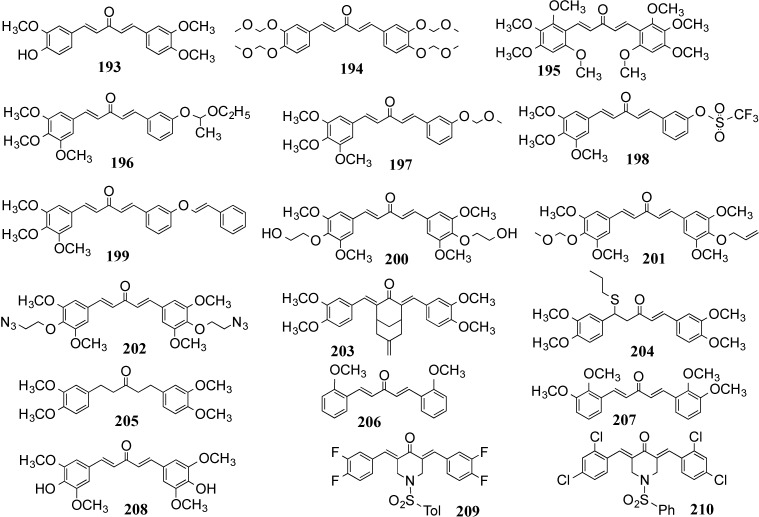
Structures of anti-cancer MACs **193**–**210**.

Yamakoshi *et al.* reported the cytotoxicity of MACs **193**–**205** (Figure 17) to the human colon cancer cell line HCT-116 [98]. SAR analysis complemented other studies by highlighting the structural motifs for *bis*-(arylmethylidene)acetone and 3-oxo-1,4-pentadiene and the degree of substitution as being important for maintaining high levels of cell cytotoxicity. Interestingly, the compound structures studied in this work suggests that the symmetry of the compounds is relatively insignificant for cytotoxicity. Zhang and co-workers prepared 26 asymmetric monocarbonyl analogs and demonstrated that five of them strongly inhibit the release of tumor necrosis factor-α and interleukin-6, while also showing much higher chemical stability than curcumin itself [28]. Thus, while the compounds are certainly suitable for use as agents against acute inflammatory disease, it remains to be seen whether the additional synthetic steps and the low-to-modest yields in some cases justifies the asymmetry. Liang’s team likewise tested a series of MACs for the anti-tumor activity and presented an SAR that implied compounds such as **206** and **207** (Figure 17) can induce tumor cell apoptosis by activating the stress mechanism of endoplasmic reticulum [99,100]. The two compounds have been reported to be under preclinical study for non-small cell lung cancer.

Other curcumin analogs, such as **30** (Figure 6) and **208** (Figure 17) also inhibited phosphorylation of STAT3 in breast and prostate cancer cells. In addition, these analogs exhibited more potent activities than curcumin on the down-regulation of signal transducer and activator of transcription (STAT3), AKT, and HER-2/neu, as well as the inhibition of cancer cell growth and migration [101,102].

Malhotra and Rawat *et al.* reported a series of novel 3,5-bis(arylidene)-4-piperidone-based symmetrical MACs and screened them for their potential anticancer activity. Among reported compounds, **209** and **210** (Figure 17) showed significant inhibition against various human tumor cell lines. Mechanism studies with the COLO 205 cell line suggests that compound **209** activates both caspase 8 and 9 and moderately activates effector caspase 3, which combined with the DNA fragmentation event suggests an apoptotic mechanism. Compound **210** delivers the characteristic annexin positive result, DNA fragmentation and caspase 3 activation. The activation of caspase 8, but not caspase 9 however, suggests an apoptosis extrinsic mechanism [103].

**Figure 18:**
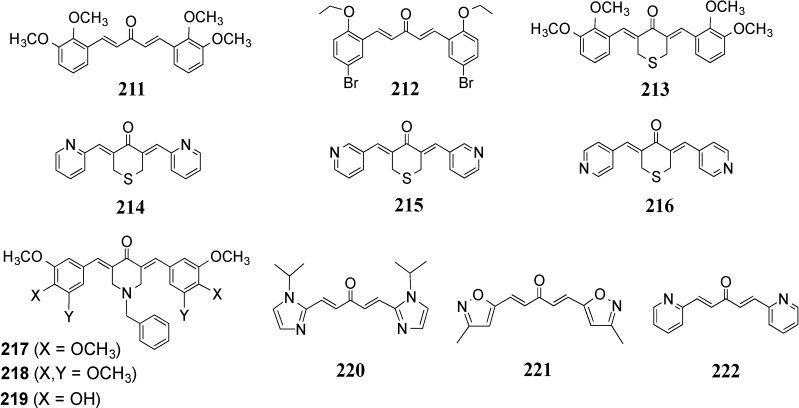
Structures of anti-cancer MACs **211**–**222**.

More recently, compound **211** (Figure 18) was subjected to HUVEC *in vitro* assay and shown to elicit anti-angiogenic activity by suppressing the downstream protein kinase activation of vascular endothelial growth factor (VEGF) via decreasing phosphorylation of AKT and p38 [104]. The same group reported that **212** displays an anti-tumor effect in the MTT cell proliferation assay using a H460 non-small cell lung cancer cell line [105].

A Guangzhou-New Jersey collaboration has reported *in vitro* activity for two series of analogs, the thiopyran-4-ones **213**–**216** [106,107] and the benzyl piperidones **217**–**219** [108] (Figure 18). The thiopyranones were tested in an MTT proliferation assay against prostate PC-3, HT-29 colon and Panc-1 cancer cell lines and shown to deliver suppressive IC_50_ values < 1 μM. All block transcriptional activity of NF-κB, modulate phosphorylation of ERK1/2 and reveal potent stimulation of apoptosis. The compounds are also uniformly 10–40 fold superior to curcumin in growth inhibition assays. In the parallel study with the benzyl piperidones **217**–**219**, PC-3, pancreas BxPC-2, HT-29, and H1299 lung cancer cell lines were probed with growth inhibition, MTT and trypan blue exclusion assays. The compounds are active with IC_50_ values < 2 μM and, similar to the thiopyranones, cause apoptosis in PC-3 cells by reduction of phosphorylation of ERK1/2 and AKT. Utilization of the benzyl moiety as a linker, the introduction of F and OCH_3_ substitution on the benzyl aromatic ring and increase of steric bulk appears to enhance the cytotoxicity. A final investigation by Samaan *et al.* [109] presented a 32-compound series of nitrogen-containing heterocyclic derivatives represented by **220**–**222** (Figure 18) as a challenge against the h-androgen independent prostate cancer cell lines PC-3 and DU-145. The analogs are reported to furnish anti-prostate cytotoxicity IC_50_ values of 50–390 nM, but no toxicity against MCF-10A normal mammary epithelial cells. The terminal 5-membered ring heterocyclic molecular class (**220**–**221**) would appear to serve as an effective bio-isostere of the now well-recognized potency and solubility enhancing pyridine series (**222** and others).

### 4.2. In Vivo Cancer Models, Tumor Growth and Regression: MACs

It is often the case that an apparently exciting *in vitro* profile proves to be ineffective in an animal model. As a result, the cell-based outcomes described in the previous two sections and those below require *in vivo* complementation. To date, a number of rodent models have been reported. One of the earliest by Shoji *et al.* involved treatment of athymic nude mice carrying MDA-MB-231 breast cancer solid tumors with EF24 (**6**) [23]. Significant anti-tumor effects were observed at 20 mg/kg with a 30% reduction of tumor weight to control. At 100 mg/kg, the tumor weight dropped to 55% without harmful side effects; namely liver, kidney and spleen toxicities were absent, and the mice experienced normal weight gain. The maximum tolerated dose (MTD) of 200 mg/kg i.v. indicated that in this setting **6** appears to be considerably more effective and substantially safer than the clinical drug cisplatin (MTD = 10 mg/kg i.p.). Perturbation of the same cell line with **6** demonstrated cell cycle arrest in the G2/M phase, an increase in intracellular ROS levels [67] post-transcriptional inhibition of the pro-angiogenic transcription factor HIF-1α, unlike curcumin, induction of microtubule stabilization in cells [87]. In a parallel study by the same group [110] coagulation factor VIIa (fVIIa) was employed as a carrier to deliver EF24 to tissue factor (TF) on the surface of the cancer cells, significantly decreasing the viability of TF-expressing MDA-MB-231 and HUVEC cells. Subsequent i.v. administration of the EF24-FFRck-fVIIa conjugate to human breast cancer xenografts in athymic nude mice leads to complexation of the conjugate to TF, endocytosis of the complex and presumed hydrolysis of the drug within the cytoplasm. The internally sequestered EF24 induces apoptosis and significantly reduces tumor size by comparison with systemically distributed and unconjugated EF24. By conjugating potent and toxic drugs to fVIIa, this targeted drug delivery system has the potential to enhance therapeutic efficacy in breast cancer and possibly other cells, while reducing toxic side effects by a combination of anti-angiogenic and anti-cancer actions.

Similar encouraging results for EF24 (**6**) have been observed in the recent past for both subcutaneous and orthotopic hepatocellular carcinoma (HCC) models [111]. DU145 prostate cancer xenografts in immunocompromised mice [112] energy metabolism and ovarian cancer metastasis models [113] ovarian carcinoma xenografts [114] and potent growth inhibition (GI_50_) of a HCT-116 human colon tumor xenograft in mice with little apparent toxicity [115,116]. As a companion to these murine studies, comprehensive pre-clinical mouse pharmacokinetics and metabolism have been performed for EF24 (**6**) by application of an LC/MS/MS assay [18]. The compound is stable in human and rat plasma at 37° for over two days and over a month at −20 °C. Protein binding in human, dog, rat and mouse plasma was >94% for all concentrations, albumin in human plasma being the primary target. EF24 pharmacokinetics in male CD2F_1_ mice were carried out at 10 mg/kg by i.v., i.p. and oral administration and best fit to a 3-compartment model. Terminal elimination half-life was 74 min accompanied by rapid absorption after oral administration, while i.v. dosing delivered a peak plasma concentration of 2.5 μM well above the *in vitro* GI_50_ value for sensitive cell lines in the NCI 60 cell line screen. Metabolism was monitored with liver microsome preparations and shown to be dominated primarily by NADPH-dependent reductive metabolism with a contribution from aromatic ring hydroxylation in the human fractions. A related liver microsome study for **7** (UBS109) reveals that reductive metabolism is also the primary deactivating mechanism, and that the primary metabolite with a single reduced C=C bond retains ~20% of the activity of **7** [19]. The high oral bioavailability of **6** (35 and 60% after i.p. and oral administration, respectively) contrasts markedly with the extremely low value for curcumin in both rodents and humans. These features, along with the potent cytotoxic activity, were interpreted to suggest that EF24 (**6**) is a promising candidate for cancer drug development. Closely related analogs of EF24 (**6**) in which the terminal aromatic rings are replaced by pyridine rings, namely EF31 (**7a**) and UBS109 (**7b**) (Figure 2), both supplement and confirm the present observations for a range of other cancer types and for inflammation as well. A comparative study examined the effects of the two analogs on human head and neck squamous cell carcinoma (SCC) Tu212 xenograft tumors established in athymic nude mice. The more soluble and more effective EF31 provided a dose response at 15 and 25 mg/kg i.p. and average AUC(0–∞) values of 3386 and 7769 h × ng/mL, respectively [12]. UBS-109 likewise delivers a dose response at 50, 100, and 150 mg/kg p.o. on SCC Tu212 xenografts, and 10 and 20 mg/kg i.p. on human pancreatic carcinoma Mia-Paca xenografts in athymic nude mice [14].

Separate investigations have examined bone mass loss in connection with metastatic breast cancer. Initially, curcumin, EF31 and UBS109 were compared for their relative effects on osteoblastogenesis and osteoclastogenesis *in vitro*. EF31 and UBS109 revealed a suppressive effect on osteoclastogenesis relative to curcumin, while UBS109 (**7b**) has a unique stimulatory effect on osteoblastic differentiation and mineralization associated with Smad signaling. **7b** also potently inhibits NF-κB, which plays a pivotal role in osteoclastogenesis [117]. In a follow-up, UBS109 (**7b**) was examined for preventive effects on bone loss induced by breast cancer cell bone metastasis [118]. Nude mice were inoculated with the corresponding cells into the head of the right and left tibia. Within a week, the mice were treated with control and UBS109 under both oral (50 or 150 mg/kg) and i.p. (10 or 20 mg/kg) administration once daily for 5 days/week for 7 weeks. X-ray diagnosis was used to ascertain remarkable bone loss in the untreated femurs and tibias. On the contrary, bone loss was prevented by both p.o. and i.p. treatment in the subject animals presumably by stimulating osteoblastic mineralization and suppressing osteoclastogenesis. The potential for human intervention resides in the possible control of breast cancer accompanied by parallel bone growth and restructuring.

A particularly challenging condition without effective remedy is pancreatic cancer. An important mechanistic feature is aberrant DNA methylation, driven mainly by DNA methyltransferases. Accordingly EF31 (**7a**) and UBS109 (**7b**) have been probed for their ability to inhibit methylation in both *in vitro* and *in vivo* settings [14]. It is known that DNMT-1 is dependent on Hsp90 for activation and stability. Treatment of two pancreatic cell lines, Mia-PaCa-2 and PANC-1, with **7a** and **7b** resulted in inhibition of Hsp90 activity through its decreased binding to DNMT-1, decreased levels of Hsp90 expression and the overexpression of three pivotal silenced genes commonly silenced in the spontaneous promotion of pancreatic cancer. Under *in vivo* conditions, Mia-Paca-2 xenografts were established in 5-week old nude mice and treated with 25 mg/kg doses of **7a** and **7b** i.v. three times weekly for 30 days. A panel of biomarkers was extracted from the tumors to show, among other things, that DNMT-1 is clearly downregulated and associated with re-expression of the silenced genes. In addition, EF31 (**7a**) and UBS109 (**7b**) potentiate the inhibition of cell proliferation by oxaliplatin and 5-fluorouracil (5FU), two agents in common anti-cancer use in the clinic. A follow-up study reveals that these two compounds also exert an increased antiangiogenic effect on pancreatic cancer both *in vitro* and *in vivo*. [119]. The unique properties of **7a** and **7b** appear to make them promising therapeutic agents for development in pancreatic cancer as single agents or as part of chemosensitized chemotherapy cocktails.

**Figure 19:**
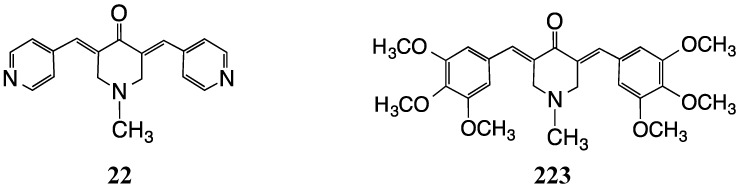
MACs **22** and **223** are effective *in vitro* against triple negative breast cancer.

Finally, two piperidines **22** (Figure 5) and **223** (Figure 19) assayed by Yadav and colleagues display the well-known inhibition of NF-κB activation complemented by substantial cytotoxicity towards MBA-MB-231, MDA-MB-468 and SkBr3 breast cancer cell lines; models for triple negative breast cancer disease [73]. Against the MBA-MB-231 cell line, the EC_50_ values are submicromolar at 0.8 and 0.3, respectively, while the two agents also cause apoptosis in 40%–45% of the SKBr3 and MDA-MB-231 cells, respectively.

In a subsequent investigation, **22** suppressed the growth of MDA-MB-468 xenograft tumors by 48% compared to control following 10 weeks of daily oral dosing (8.5 mg/kg) [120]. The compound also evidenced anti-angiogenic properties *in vitro* by inhibiting 46% of HUVEC cell migration and the induction of these cells to form tubular networks. Thus, **22** presents potent proapoptotic and anti-angiogenic assets *in vitro* and *in vivo*, suggesting it to be a potential and promising remedy for treatment of ER-negative breast cancer. Other *in vivo* rodent studies not specifically reviewed here are likewise encouraging that the wide-ranging MAC family of anti-inflammatory and anti-cancer agents may well be able to address the corresponding debilitating conditions in humans sometime in the future [8,15,16,17,67,68,90,97,110,111,114,118].

## 5. Bacterial Growth

Curcumin has received far less attention as inspiration for a potential antibacterial agent by comparison with its influence on the development of cancer blockers. This is surprising given that each year in the U.S. at least 2 million people become infected with bacteria resistant to antibiotics causing an annual death toll of approximately 23,000 arising directly from the infections [121]. The situation has become sufficiently dire that during the fourth week of September, 2014, President Obama has directed the CDC to create a national plan to control the situation [122]. Importantly, since inflammation follows infection as described in the previous section, were curcumin and mimics to become effective antibiotic agents, they offer an advantage in the ability to mediate simultaneously both bacterial titer and the inflammatory response. Early reports catalog curcumin’s blockade of various bacteria [123,124]. One recent study applied the molecule and its more soluble conjugate, curcumin glucoside, to two Gram-positive (*Bacillus cereus*, *Staphylococcus aureus*) and two Gram-negative (*Escherichia coli* and *Yersinia enterocolitica*) pathogenic bacteria. All four exhibited moderate growth inhibition in response to treatment by both molecules [125]. In a related study [126], it was noted that curcumin induces filamentation in Gram-positive *Bacillus subtilis* (strain 168), suggesting the molecule to be able to modulate bacterial cytokinesis (cell division to daughter cells). At the molecular level, the process is regulated by the assembly dynamics of FtsZ protein protofilaments, a protein aggregate that assembles into a ring at the future midpoint of bacterial cell division [127]. FtsZ is a prokaryotic homologue of the eukaryotic protein tubulin that regulates the function and trafficking of microtubules. Curcumin was shown to inhibit assembly *in vitro*, increase GTPase action and bind to the protein accompanied by perturbation of secondary structure. Such actions, lethal to the bacteria, provide a provocative mechanistic hypothesis for the action of the drug. These observations were followed by a docking study exploring the interaction of curcumin with the catalytic cores of the *E. coli* and *B. subtilis* FtsZ proteins [128] The tentative location of the binding site, supported by bacterial mutagenesis, was proposed as an opportunity to access novel inhibitors by structure-based molecular design [129,130,131].

Three interesting reports have explored MACs as candidates for bacterial control. An early exploration presented 40 compounds in the a, b and c classes shown in Figure 4 and compared their actions as monitored by zones of inhibition against seven multi-drug resistant bacteria containing both Gram-positive and Gram-negative examples [132]. A few members of each inhibitor class are very similar to the action of curcumin, but all are less potent than the β-lactam antibiotic ampicillin. Encouraging, however, most were found to be active against *Enterobacter cloacae*, an organism that is clinically resistant to ampicillin. On average, the acyclic a-class was claimed to be overall most effective, though the differences would appear to be small. Only one substitution offered a sizeable *ortho*-substituent (Br). This member of each of the three classes proved to be inactive, perhaps due to steric distortion (*cf.* Figure 4).

**Figure 20:**
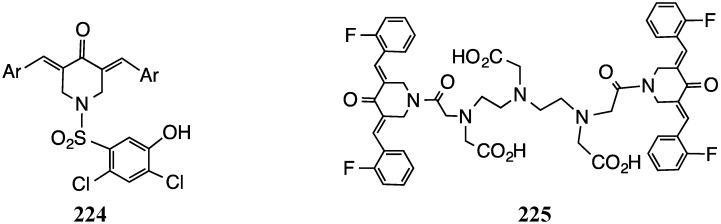
MACs **224** and **225** block the growth of multiple bacterial strains *in vitro*.

The second study prepared 15 piperidones and screened them against *Salmonella typhi*, *Vibrio cholerea*, *Escherichia coli* and *Staphylococcus aureus* [133] Several compounds produced significant zones of inhibition (e.g., **224**, Figure 20) for the first two bacteria superior to the action of both curcumin and penicillin. More modest activity was observed for the other analogs against all four bacteria.

Finally, a very recent investigation explored the effects of EF24 (**6** and **14**, Y = C-F, X = NH) and its dimer **225** (EF_2_DTPA) against Gram-negative (*Escherichia coli*) and Gram-positive (*Enterococcus faecalis*, *Staphylococcus aureus*) bacteria [134]. Both classes experience direct bacterial growth suppression. Additional experiments demonstrated that the compounds do not influence bacterial uptake or localization in dendritic cells. Although the antibacterial potencies of the synthetics are considerably lower than that of traditional antibiotics, the authors suggest that such substances might be useful in combination with other chemopreventive agents in appropriate situations.

Although the literature supporting the results discussed in this section have not explored the FtsZ connection, they clearly illustrate that MACs carry antibacterial activity and, in some cases, are effective against highly resistant bacteria. Considering the structural insights derived from examining the FtsZ-curcumin complexes [124]. it is likely that a similar evaluation of modeled FtsZ-MAC complexes would lead to suggestions for compound modification and drive investigations to confirm the prospective binding and lead to novel antibacterial candidates.

## 6. Tuberculosis

Tuberculosis (TB) is an airborne disease caused by the contagious and ancient bacterium *Mycobacterium tuberculosis* (*M. tuberculosis)*. TB is considered latent when the *M. tuberculosis* infection is present but the immune system is able to prevent active multiplication. If the immune system is unable to prevent bacterial growth, TB becomes active and can be fatal, if untreated. About one third of the world’s population is infected with the microorganism. In 2012 there were nine million new cases and 1.3 million TB-related deaths around the world [135]. Not surprisingly, TB is the leading killer of people infected with immune compromised HIV, making TB one of the most deadly diseases in the world. Currently, ten drugs are approved by the FDA to treat active TB [136] with a combined success rate reported to be 87% in 2011 [137]. While current drugs are highly effective against the TB bacteria, drug-resistant strains are becoming more prevalent especially in less developed countries. Contemporary treatment methods for drug-resistant TB are less effective and 50 to 200 times more expensive than treatment for classic cases of TB [138]. As a consequence, discovery of alternative drugs is of high importance.

Curcumin was reported to inhibit *M. tuberculosis* growth as early as the mid-twentieth century, making it an underexplored but promising drug candidate against TB. In 1949 Schraufstatter and colleagues reported that curcumin is able to completely block *M. tuberculosis* growth *in vitro* at a dilution of 1:40,000 [139] A recent review by Marathe and co-workers argues that curcumin may serve as an inhibitor against efflux pumps in TB bacteria. Efflux pumps mediate the efflux of antibiotics, drugs and other classes of compounds. Several studies indicate that efflux pumps are behind the survival mechanism for resistant *M. tuberculosis* [140,141,142]. Another investigation by Dube and colleagues used computational models to focus on a specific domain of an essential enzyme for *M. tuberculosis* (the BRCT domain) known as *M. tuberculosis* enzyme (*Mtu*LigA) [143]. *Mtu*LigA is essential and unique to TB bacteria, making it an intriguing drug target. Computational models suggested that a curcumin derivative, monoacetylcurcumin, binds to the BRCT domain, but no experiments were performed to confirm inhibition of *Mtu*LigA by curcumin or its analogs. Takeuchi and co-workers conducted *in vitro* experiments to study the inhibitory activity of curcumin derivatives against DNA polymerase λ [144], which also contains the BRCT domain as part of its active site. Curcumin itself demonstrated strong inhibition of DNA polymerase λ (IC_50_ 7.0 μM), while its monoacetyl derivative proved to be nearly twice as effective (IC_50_ 3.9). Agrawal *et al.* reported that demethoxycurcumin (**2**) can block M. tuberculosis H37Rv strain with a minimum inhibitory concentration (MIC) of 200 μg/mL (5.8 × 10^−4^ M) [145]. Of four synthetic derivatives of **2**, two analogs exhibited values of 125 and 8 μg/mL MICs. This compares with a rifampicin control at 2 μg/mL. The latter most active compound carries an R = OCH_2_-CH=CH-CO_2_C_2_H_5_ *p*-substitution, while the former lacking a conjugated ester (R = OCH_2_CO_2_C_2_H_5_) is 16-fold weaker. Michael addition may therefore play an important role in the mechanism of such compounds. Bairwa *et al.* have recently reviewed a broader set of similarly substituted keto-enol curumin derivatives and drawn a similar conclusion [27]. Finally, while curcumin shows promising anti-TB activity *in vitro*, it fails to show similar effectiveness *in vivo* [146]. One wonders if the curcumin derivatives mentioned here would show the same fate.

**Figure 21:**
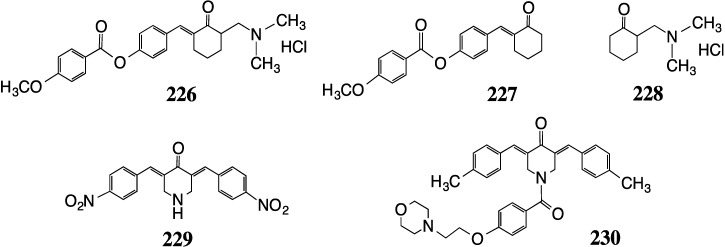
Structures of various MACs serving as *in vitro* antimycobacterial agents against TB (**226**–**230**).

As previously stated, replacement of curcumin’s keto-enol functionality with a single carbonyl group may serve as a way to achieve greater stability and perhaps enhanced performance. MACs and related analogs have not been thoroughly explored at the time of writing, implying a potentially important direction for this class of molecules as anti-tubercular agents. Dimmock and co-workers have addressed this problem by combining the MACs architecture with a Mannich base and evaluating the compounds *in vitro* for antimycobacterial properties against *M. tuberculosis* H_37_R_v_ [147]. At 12.5 ug/mL, the Mannich bases achieved 99% inhibition, while a related series of unsaturated ketones blocked microorganism growth by only 21%–66%. Two of the bases were both exceptionally active with MIC values of 0.39 ug/mL (**226**, second structure not shown), which compares very favorably with rifampin (0.16 uM/mL). A follow-up evaluation on **226** sought to understand the origin of the high activity by preparing the partial structures **227** and **228** (Figure 21) [148]. Compound **227** proved to be essentially inactive, but **228** gave a moderate MIC value of 12.5 uM obviously highlighting the N(CH_3_)_2_ moiety. A probe of toxicity against Vero cells showed selectivity indices (IC_50_/MIC) for **226** and **228** to be 21 and 0.004, the former considered to signal a lead. Thus, enone **226** was evaluated against seven strains of *M. tuberculosis* including multidrug resistant (MDR) and extensively drug-resistant (XDR) strains to deliver MIC values across the board very similar to that of *M. tuberculosis* H_37_R_v_. Accordingly, the drug-resistant strains do not evidence cross resistance to Mannich base **226**, which appears to exert a different mode of antimycobacterial action relative to that by a panel of other TB drugs.

A separate investigation focused on the preparation of a series of piperidones, which showed excellent *in vitro* antimycobacterial properties against *M. tuberculosis* H_37_R_v_ [149]. Among others, **229** and **230** are 100% inhibitory at 6.3 μg/mL with MICs at 0.2 and 0.8 μg/mL, respectively. In addition, the compounds are generally well tolerated in mice as judged by toxicity and neurotoxicity screens. Oral and ip dosing of a selection of the piperidones in rats likewise failed to reveal neurotoxicity, although some swelling and stimulated respiration in rat liver mitochondria was observed. The two effects seem to be disconnected. Missing from this report is a study of the effects of the synthetic agents in animals infected by *M. tuberculosis*.

More recently, EF24 (**6**), EF31 (**7a**) and UBS109 (**7b**) were examined for *in vitro* growth inhibition of both *M. marinum* and *M. tuberculosis*, including rifampicin-resistant strains [150]. The compounds deliver IC_50_ values in the 4-25 μM range, considerably lower than curcumin. An important mechanistic finding of this work is that the anti-mycobacterial action of the MACs appears to be dependent on Michael addition chemistry.

**Figure 22:**
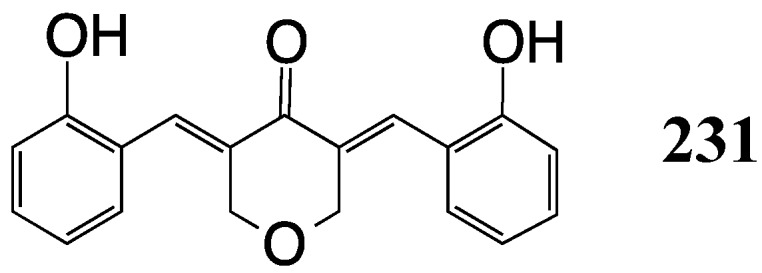
A heterocyclic MAC that blocks autophagy (**231**).

We finish with a caveat. It has recently been shown that in a Drosophila model host system, host autophagy activation is essential for antimicrobial action against *M. tuberculosis* in adult flies [151]. Such activation would appear to be required for homeostatic control during chemotherapy against the TB microorganism. Blocking autophagy with antibiotics, *i.e.*, with curcumin derivatives or MACs, might well compromise the host autophagy in the successful delivery of drug to *M. tuberculosis* during treatment. One of our MACs, EF25 (**231**, Figure 22), has recently been shown to block autophagy and contribute to cell death in a xenograft-based hepatocellular carcinoma study [152]. It remains to be seen whether the inhibition of autophagy by MACs in a cancer model would translate adversely to a TB model in which the drug would eliminate *M. tuberculosis* but simultaneously counter the effect by providing a safe haven for the microorganism during chemotherapy.

## 7. Alzheimer’s Disease

Alzheimer’s Disease (AD) is a progressive neurodegenerative disorder that most often progresses to dementia for the aging population. It is estimated that 36 million people are afflicted by dementia worldwide with eight million new cases annually. The most common cause of dementia is AD accounting for 60%–70% of all such cases. While this obviously takes an irreversible toll on the recipient, AD also exacts a devastating price for families and caregivers [153]. The condition can be characterized in part by amyloid β (Aβ) aggregation, inflammation and oxidative damage in the brain. Unfortunately, not only are the pathological mechanisms not fully understood, no chemotherapeutic agents yet exists to either ameliorate or eliminate the illness. Curcumin, with its anti-oxidative and anti-inflammatory properties, is an under-explored but promising candidate for AD treatment.

Recent studies indicate that curcumin or its analogs may be able to effectively address a wide array of AD symptoms [154]. In an early study conducted in 2004, curcumin was shown to bind to and inhibit the formation of Aβ oligomers and fibrils. *In vitro* experiments showed that curcumin inhibits Aβ aggregation more effectively than the over-the-counter NSAIDs ibuprofen and naproxen, anti-inflammatory drugs known to reduce amyloid formation [155]. *In vivo* experiments from the same study disclosed that curcumin injected peripherally crosses the blood-brain barrier, binds to plaques and reduces levels of amyloid aggregation in mice with advanced amyloid accumulation. The outcome is consistent with the hypothesis that fibrils and plaques are the cause of AD, and that they can be diminished *in vivo*. Accordingly, the authors suggested that curcumin should be taken to the clinic. A number of trials have been performed, and at the time of this writing (October, 2014) seven are underway [156,157]. Unfortunately, none have revealed a decisive counter to AD. In another subsequent study, a seven-day curcumin treatment regimen reduced levels of plaque by 30% in a mouse model of AD. The regimen also significantly reversed structural dendritic abnormalities. Curcumin was found to have limited effectiveness in crossing the blood-brain barrier, but results indicate a high affinity for Aβ that may contribute to the inhibition of amyloid formation [158]. A molecular approach to understanding how small molecules interact with amyloid-β has generated X-ray structures of four compounds complexed with amyloid-like segments, the small molecules lying within cylindrical cavities running along β-spines of the fibers [159]. The electron density for curcumin at low resolution does not permit modeling in atomic detail, but it strongly suggests that the long axis of the molecule lies parallel to the fiber axis as it does for the other ligands examined. The work proposes a general pharmacophore for Aβ aggregates suggesting that mixtures of compounds may be necessary in any future treatment to address the collection of polymorphic aggregates that appear to characterize the amyloid fibrils. A separate analysis sought to determine the tautomeric form of curcumin binding to Aβ aggregates [160]. It was concluded that the keto-enol form is the dominant form associated with the latter and that targeting the enolization process might offer a target for design of novel amyloid-binding agents. However, observation of the enol form in the amyloid context is entirely consistent with detailed examination of curcumin itself demonstrating the overwhelming presence of the keto-enol form [161], apparently eliminating the possibility to target tautomerization.

While curcumin shows tantalizing effectiveness against AD, its low bioavailability and stability as discussed in the Introduction poses problems for its potential as a drug. As emphasized in previous sections, one way to bypass such characteristics is pursuit of curcumin analogs with more suitable biophysical and physicochemical properties.

**Figure 23:**
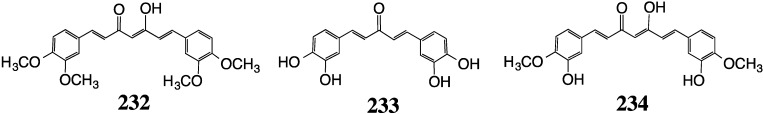
Potential oligomerization inhibitors against the Aβ peptide and fibrils (**232**–**234**).

Orlando and co-workers [162] recently screened a library of curcumin derivatives and MACs with a novel ELISA-based assay that quantifies oligomeric Aβ peptide and distinguishes the oligomeric aggregate from fibrils. The work identified methylated curcumin **232** (Figure 23) as an oligomerization inhibitor (IC_50_ 0.15 μM) with 6–7 fold improvement over curcumin (IC_50_ 1.0 μM). MAC **233** (IC_50_ 0.8 μM) likewise proved nearly equivalent to curcumin by assay. In addition, a preliminary SAR provides some clues for future workers: (1) A least one enone group in the spacer between aryl rings is necessary to observe anti-Aβ aggregation; (2) Unsaturation in the linker is also required for inhibition; (3) Both methoxy and hydroxyl substitutions at the *m*- and *p*-positions elicit de-aggregation, a positive combination being a curcumin reversal (*m*-OH, *p*-OMe, **234** IC_50_ 0.6 μM). It is interesting that the MAC variation **233** seems to offer little in the way of potency as it does for cancer and inflammation. A more elaborate SAR is likely to uncover improved and perhaps highly potent MAC candidates at this early stage of discovery.

**Figure 24:**
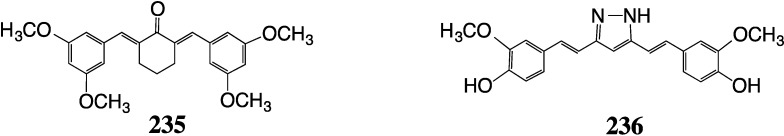
A potential Aβ peptide blocker (**235**) and an *in vitro* chloroquine-sensitive (CQ-S) and chloroquine-resistant (CQ-R) *P. falciparum* MAC antimalarial (**236**).

Oxidative stress is believed to be a major factor in the progression of AD by causing neurological degeneration and death [163]. An effective antioxidant against AD-related oxidative stress is an attractive strategy. An early study examined the antioxidant capacities of curcumin and (2E,6E)-2,6-Bis(3,5-dimethoxybenzylidene)cyclohexanone **235** (MCH, Figure 24) [164]. The compound is relatively stable by comparison to curcumin and shows enhanced anti-oxidative performance *in vitro*. Rat pheochromocytoma PC12 cells, a model for neurological cells under Aβ oxidative stress, were found to be protected from H_2_O_2_ injury by maintaining endogenous antioxidant enzyme activity, scavenging for reactive oxygen species and shielding cells from H_2_O_2_-induced cytotoxicity and apoptosis. *In vivo* experiments for MCH were not conducted, but the *in vitro* results suggest a promising avenue for further exploration.

## 8. Malaria

Malaria is a serious infectious disease characterized by fevers, chills and flu-like illness. It is transmitted by *Anopheles* mosquitos infected with five known species of *Plasmodium* parasites, which include *P. falciparum*, *P. vivax*, *P. malariae*, *P. ovale* and *P. knowlesi*. Three of the five species have been reported to exhibit drug-resistant behavior, while *Plasmodium* parasites are gaining resistance against an increasing number of different anti-malarial drugs. With the growing issue of drug-resistance, it is of paramount interest to search for alternative anti-malarial drugs that are effective and inexpensive.

Several studies indicate that curcumin and its analogs may well serve as effective anti-malarial agents. According to Reddy and co-workers, curcumin delivers significant anti-malarial activity both *in vitro* and *in vivo* [165]. Curcumin inhibits chloroquine-resistant *Plasmodium falciparum* in culture in a dose-dependent manner (IC_50_ 5 μM). In the *in vivo* experiments, curcumin was orally administered to *P. berghei*-infected mice in a dose known to be safe for humans. The drug treatment resulted in an 80%–90% reduction in blood parasitemia, and curcumin treated mice experienced an overall survival rate of 29% compared to 0% for the non-treated control. In another study by Mishra and colleagues, *in vitro* experiments were performed to examine the effectiveness of curcumin and its analogs against red blood cell cultures containing either chloroquine-sensitive (CQ-S) or chloroquine-resistant (CQ-R) *P. falciparum* [166]. The agent was found to significantly inhibit the growth of both CQ-S (IC_50_ 7.9 μM) and CQ-R (IC_50_ 29.6 μM) in red blood cell cultures. Several structural modifications of curcumin were also tested against both CQ-S and CQ-R, and many exhibited enhanced inhibitory activity. A pyrazole analog **236** (Figure 24) demonstrated the most effective inhibitory performance among the compounds tested (CQ-S (IC_50_ 0.48 μM) and CQ-R (IC_50_ 3.9 μM)). The authors speculated that the improved anti-malarial activity of 226 arises from increased cellular uptake and retention as well as greater inhibitory potency against the target itself.

**Figure 25:**
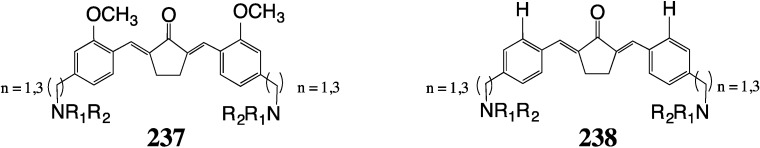
Potent *in vitro* CQ-R and CQ-S inhibitors of the *P. falciparum* parasite (**237**–**238**).

A more recent study evaluated the antimalarial activity of MACs against the two strains of *P. falciparum*, and found that the compounds exhibit significant anti-malarial behavior in an *in vitro* setting [167]. Twenty different MACs were tested against *P. falciparum* cultures and six of them were found to provide excellent inhibition of both CQ-R and CQ-S (**237**, **238**, IC_50_ < 1 μM, Figure 25). Considering the increased bioavailability and stability of MACs as well as their enhanced anti-malarial abilities, this class of molecules offers an intriguing direction for further molecular design opportunities.

## 9. Summary

A host of MACs has shown potent anti-tumorigenic activity when tested by *in vitro* and *in vivo* models spanning a wide range of cancers and inflammatory conditions. Although a number of structure-activity relationship (SAR) studies reveal useful correlations within a limited structural set of analogs [168], a truly broad analysis has yet to be achieved. Likewise, few of the reported structure activity studies make an attempt to deal with the subtle impact of 3D geometry as implied by Figure 4. To be sure, the size and electronegativity of substituents on the terminal aromatic rings (Figure 10) and the rigidity of the spacers between these rings may influence the *in vitro* biological actions of MAC’s. In addition, four other features have surfaced as advantageous elements for overall improved activity: (1) A doubly conjugated carbonyl is generally more potent than a single enone; (2) Double or triple methoxy substitution on the terminal aromatic rings, often accompanied by an aromatic OH group, frequently provides increased activity; (3) Terminal pyridine rings furnish both superior potency and solubility; (4) A piperidone ring at the center of a MAC not only imparts solubility, but also creates the opportunity to append extensions through an amide-like linker with additional beneficial properties. Assuming protein binding sites can tolerate such structural modification, specific and selective targeting might be an option. One example illustrated by *in vivo* testing involved the coupling of EF24 (**6**) to coagulation factor VIIa followed by protein-protein interaction with tissue factor leading to endocytosis and targeted delivery of the drug to the cytoplasm of MDA-MB-231 breast cancer cells. To date, this has not yet blossomed into a predictive and fruitful pathway for investigation, most likely because there are no X-ray structures of the corresponding ligand-protein complexes at present. It goes without saying that the same situation exists for the parent template with no single macromolecule-curcumin solid-state structure having been solved. In spite of the paucity of well-determined MAC-protein structural models, a growing number of investigations highlight that MACs are superior to curcumin *in vitro* by a 10–20 fold potency gain across a wide range of cancer cell lines, inflammation models and against numerous signaling proteins and transcription factors. Likewise, murine-based *in vivo* studies demonstrate clearly that MACs with appropriate pharmacokinetic and ADME properties (Absorption, Distribution, Metabolism and Excretion) can be conceived, prepared and tested for potential relief of human cancer, inflammatory diseases and other conditions. Similarly, by virtue of their improved physical properties, MACs have successfully demonstrated good PK profiles in mice and promoted tumor regression in cancer xenografts as well as orthotopic models *in vivo*; almost always more efficacious by comparison to curcumin. Though the literature is not yet deep, chemical and biological efforts to create MACs with the potential to relieve TB, Alzheimer’s, malaria and other maladies is underway. Based on the available data, it would appear that many of the underlying biochemical mechanisms controlling the latter conditions are common. One can be hopeful and optimistic that new developmental drugs might be capable of cross-treating any number of them.

## Abbreviation

MACs: monocarbonyl analogs of curcumin
Aβ: amyloid β
AD: Alzheimer’s Disease
AKT: protein kinase B
ALR: aldose reductase
AP-1: the activator protein 1
ATP: adenosine triphosphate
COX-2: cyclooxygenase
DNMT: DNA methyltransferase
ERK: extracellular-signal regulated kinase
HIF: hypoxia inducible factor
HSP: heat shock protein
HUVEC: human umbilical vein endothelial cells
IL: interleukin
i.p.: intraperitoneal
i.v.: intravenous
JNK: c-Jun N-terminal kinase
MAPK: mitogen-activated protein kinase
MID: minimum inhibitory concentration
MTD: maximum tolerance dose
MTT: 3-(4,5-dimethylthiazol-2-yl)-2,5-diphenyltetrazolium bromide
LOX: lipoxygenase
LPS: lipopolysaccharide
NADPH: nicotinamide adenine dinucleotide phosphate
NCI: National Cancer Institute
NF-κB: nuclear factor κB
NO: nitric oxide
NSAID: nonsteroidal anti-inflammatory drug
ODC: ornithine decarboxylase
p38: stress-activated protein kinase
PGE: prostaglandine E
p.o.: per os (by mouth)
RAW264.7: mouse macrophage-like cell line
ROS: reactive oxygen species
SAR: structure-activity relationship
STAT: signal transducer and activator of transcription
TB: tuberculosis
TF: tissue factor
TNF: tumor necrosis factor
TPA: 12-*O*-tetradecanoyl-13-acetate
VEGF: vascular endothelial growth factor
VHL: von Hippel-Lindau
